# Plasmalogens Mediate the Effect of Age on Bronchodilator Response in Individuals With Asthma

**DOI:** 10.3389/fmed.2020.00038

**Published:** 2020-02-14

**Authors:** Joanne E. Sordillo, Sharon M. Lutz, Rachel S. Kelly, Michael J. McGeachie, Amber Dahlin, Kelan Tantisira, Clary Clish, Jessica Lasky-Su, Ann Chen Wu

**Affiliations:** ^1^PRecisiOn Medicine Translational Research (PROMoTeR) Center, Department of Population Medicine, Harvard Medical School and Harvard Pilgrim Health Care Institute, Boston, MA, United States; ^2^Channing Division of Network Medicine, Brigham and Women's Hospital, Harvard Medical School, Boston, MA, United States; ^3^The Broad Institute, Cambridge, MA, United States

**Keywords:** bronchodilator response (BDR), metabolites, age, interaction, plasmalogen

## Abstract

**Background:** Asthma is known to display different phenotypes across the life-course, suggesting that age related changes are particularly relevant to understanding asthma pathogenesis and remission. We have previously demonstrated that a lung function phenotype associated with asthma, bronchodilator response, is reduced with age, at rate of 0.24 percent per year.

**Methods:** In this study, we interrogated the serum metabolome, to determine whether circulating metabolites mediate age-related changes in bronchodilator response (BDR) for individuals with asthma. We used data on 295 participants from the follow-up phase of the CAMP clinical trial (age 12.2–25.9 years; mean BDR of 8%, standard deviation 7%). Using a counterfactual framework, we analyzed over 500 pareto-scaled metabolites using mediation analysis to identify indirect effects of age through potential metabolite mediators.

**Results:** There was a significant indirect effect of age on BDR through 4 plasmalogens (C36:1 PC and related metabolites) (Indirect Effect Beta = −0.001, *p* = 0.006).

**Conclusions:** Our findings suggest that plasmalogens may contribute to age-related asthma phenotypes, and may also serve as potential pharmacologic targets for enhancement of lung function in individuals with asthma.

**Trial Registration:** This work uses data from the previous clinical trial of asthma, the Childhood Asthma Management Program (CAMP), registered at ClinicalTrials.gov, # NCT00000575.

## Introduction

Asthma is known to display different phenotypes across the life-course, suggesting that age related changes are particularly relevant to understanding asthma pathogenesis and remission (1). We previously demonstrated that bronchodilator response (BDR), which is associated with asthma, is reduced with age, at rate of 0.24 percent per year (2). The biological mediators of this age-related change are not yet understood. The metabolome may be a key source of intermediary biomarkers that contribute to clinical phenotypes in asthma, especially given that metabolites directly reflect alterations in host biochemical pathways. In fact, metabolite profiles have been associated with lung function outcomes in children with asthma (3). In this study, our objective was to interrogate the serum metabolome to determine whether circulating metabolites mediate age-related changes in BDR in children and young adults with asthma.

## Methods

To estimate the effects of metabolite mediators, we used data from the Childhood Asthma Management Program (CAMP), a multi-center, randomized, double-masked, clinical trial designed to determine the long-term effects of treatment with budesonide, nedocromil, or a placebo for mild to moderate asthma in children (4). A total of 1,041 children, 5–12 years of age, were enrolled in the trial between December 1993 and September 1995. Inclusion was restricted to children with a history of chronic asthma who, during a 28-day run-in period, had asthma symptoms or a low morning peak flow on 8 or more days; inclusion also required a methacholine challenge with a concentration of 12.5 mg per milliliter or less that resulted in a reduction in FEV_1_ by at least 20% [since airway responsiveness is determined by the provocative concentration of methacholine required to reduce the FEV1 by at least 20% (methacholine PC20), with higher values indicating less airway responsiveness]. Participants attended 3 clinical trial “visits” per year and treatment was continued for a mean of 4.5 years. Following the end of the treatment component of the trial in 1999, asthma care was transferred to each participants' health care provider. Over 85% of the original 1,041 participants participated in the 4 month transition phase (2 visits) following the trial, and 80% participated in all three phases [the treatment phase, the transition phase, and the post-trial follow-up phase (1–4 visits per year for 13 years)]. Metabolomics and bronchodilator response data from participants (*N* = 295) from the post-trial follow-up phase were used in this analysis. The CAMP study was approved by the institutional review board of Partners Healthcare (Partners Human Research Committee; Protocol#: 1999-P-001549/29), by all eight CAMP clinical centers, and by the CAMP Data Coordinating Center. Each child's parent or guardian signed a consent statement and the clinics obtained each child's assent.

Metabolomic profiling was performed on serum samples in CAMP (see Supplementary Methods). Features were analyzed as measured LC-MS peak areas, and log-transformed and *pareto* scaled prior to analysis. After QC and data processing a total of 501 named metabolites (see Supplemental Table 1) were identified by matching measured mass to charge ratio and retention time with authentic reference standards.

Lung function was measured by spirometry using a Survey Tach Spirometer (Warren E. Collins; Braintree, MA) in accordance with the American Thoracic Society recommendations. Prior to assessment, the children were told to withhold short-acting bronchodilators for at least 4 h. Spirometric maneuvers were conducted with the children seated and wearing a nose clip. The measurements were calibrated for gender, age and height according to reference values. After completing baseline spirometry, the children were given 200 μg (2 puffs) of an albuterol pressurized metered-dose inhaler (pMDI) using a spacer device. Spirometry was repeated after 15 min. BDR was defined as the best forced expiratory volume in the 1st second (FEV_1_) post-bronchodilator minus FEV_1_ pre-bronchodilator, divided by FEV_1_ pre-bronchodilator (expressed as a percentage).

For each individual metabolite mediator, we fit a linear regression model for bronchodilator response as the outcome and age as a predictor adjusting for the metabolite mediator and race, sex, treatment group and clinical site. We used a counterfactual framework for mediation analysis to estimate the indirect effects of age on BDR adjusting for race, sex, treatment group, and clinical site (5, 6). This method is implemented in the *medflex* package in *R* (7). We considered indirect effects at *p* < 0.05 as potential metabolite mediators of age on BDR.

## Results

Participant characteristics are shown in Table 1. Participants were 64% male, and the majority were of white race/ethnicity (72% white, 12% black, 7% Hispanic, and 9% other). Participants ranged in age from 12.2 to 25.9 years. The mean BDR was 8.0% and with a standard deviation of 7.0%. The top metabolite mediators were all phosphatidylcholine plasmalogens as shown in Table 2. Our results suggest that these metabolites may mediate the association of age with BDR (i.e., a portion of the reduction in BDR over time may be accounted for by age-related shifts in plasmalogen levels). These phosphatidylcholine plasmalogens were all highly correlated with one another (spearman *r* = 0.77–0.98).

**Table 1:** Characteristics of CAMP participants with metabolomics data in the post-trial follow-up phase (total *N* = 295).

**Characteristic**		***n***	**%**
Sex	Male	189	64.1%
	Female	106	35.9%
Race	White	213	72.2%
	Black	35	11.9%
	Hispanic	21	7.1%
	Other	26	8.8%
Treatment group	Budesonide	78	26.4%
	Nedocromil	83	28.1%
	Placebo	134	45.4%
Age at blood sample	Mean (SD) [range]	16.8 (2.9)	[12.2, 25.9]
% BDR at blood sample	Mean (SD) [range]	8.0 (7.0)	[−14, 49]

**Table 2:** Serum metabolite mediators for the association of age and bronchodilator response^*^.

**Metabolite**	**Average indirect effect of age through metabolite (beta)**	***P*-value**
C36:1 PC plasmalogen	−0.001	0.006
C36:3 PC plasmalogen	−0.002	0.007
C36:2 PC plasmalogen	−0.002	0.01
C36:4 PC plasmalogen	−0.001	0.03

* *Models adjusted for race, sex, clinical site, and treatment group*.

We also looked at correlation patterns within our metabolite data, and determined that phosphatidyl ethanolamine (PE) metabolites and sphingomyelin (SM) metabolites are correlated with circulating PC plasmalogens. [Our main plasmalogen of interest (C36:1 PC plasmalogen), was correlated with PE metabolite levels (Spearman *r* = 0.71 for C38:2 PE, *r* = 0.74 for C36:2 PE plasmalogen) and with SM metabolites (Spearman *r* = 0.79 for C16:0 SM, *r* = 0.74 for C22:1 SM)]. However, these PE and SM metabolites were not mediators of the association of age with bronchodilator response (*p* > 0.6 for all indirect effects).

## Conclusions

Plasmalogens are biologically plausible mediators of lung function responses, given their potential to alter the structural properties of lung surfactants (8). Plasmalogen levels are reduced following exposures known to exacerbate asthma (i.e., ozone) (9) and have been associated with a range of adverse respiratory health outcomes, including bronchopulmonary dysplasia in infants (10) and COPD in adults (11). Antioxidative effects of plasmalogens have been studied extensively both *in vitro* and *in vivo* (12), and may have relevance for bronchodilator response in asthma. Lastly, plasmalogens are biomarkers of the normal aging process (13), which further strengthens the evidence for these metabolites as age-related mediators of physiological responses.

Our study is the first to identify plasmalogens as possible mediators of age-related changes in lung function in individuals with asthma. Our findings suggest that plasmalogens may contribute to age-related asthma phenotypes, and may also serve as a potential pharmacologic target for enhancement of lung function in individuals with asthma.

## Data Availability Statement

The datasets generated for this study are available on request to the corresponding author.

## Ethics Statement

The CAMP study was approved by the institutional review board of Partners Healthcare (Partners Human Research Committee; Protocol#: 1999-P-001549/29), by all eight CAMP clinical centers, and by the CAMP Data Coordinating Center. Written informed consent to participate in this study was provided by the participants' legal guardian/next of kin.

## Author Contributions

JS, SL, and AW contributed to the design and conception of the mediation analysis. SL provided technical statistical support. SL and JS conducted the analysis and wrote the manuscript. MM, AD, KT, RK, and JL-S contributed meaningful edits and assisted in writing the manuscript. CC contributed to data generation and provided technical support.

### Conflict of Interest

The authors declare that the research was conducted in the absence of any commercial or financial relationships that could be construed as a potential conflict of interest.
