# Supplementary material for: Plasmalogens Mediate the Effect of Age on Bronchodilator Response in Individuals With Asthma
Source: Front Med (Lausanne). 2020 Feb 14;7:38. doi: 10.3389/fmed.2020.00038 (PMC7034309; doi:10.3389/fmed.2020.00038)
Supplement: Supplementary file 1 [file Data_Sheet_1.docx]

**I. Supplementary Information**

***Metabolomic Profiling***

Metabolomic profiling was performed on serum samples in CAMP. Blood was shipped to the sample repository to the Broad Institute (Cambridge, MA, USA) on dry ice for metabolomic profiling. Samples were thawed on ice for sub-aliquoting for each of the metabolomic methods, and then re-frozen on dry ice and stored at -80C until analysis.

Four complimentary liquid chromatography tandem mass spectrometry (LC-MS) methods were used. (i) Hydrophilic interaction liquid chromatography (HILIC) analyses of water soluble metabolites in the negative ionization mode (HILIC-neg) were conducted using an LC-MS system comprised of an AQUITY UPLC system (Waters; Milford, MA and a 5500 QTRAP mass spectrometer (SCIEX; Framingham, MA)^1^. (ii) HILIC analyses of water soluble metabolites in the positive ionization mode (HILIC-pos) were conducted using an LC-MS system comprised of a Shimadzu Nexera X2 U-HPLC (Shimadzu Corp.; Marlborough, MA) coupled to a Q Exactive hybrid quadrupole orbitrap mass spectrometer (Thermo Fisher Scientific; Waltham, MA)^2-5^.(iii) Positive ion mode analyses of polar and non-polar plasma lipids (C8-pos) were conducted using an LC-MS system comprised of a Shimadzu Nexera X2 U-HPLC (Shimadzu Corp.; Marlborough, MA) coupled to a Exactive Plus orbitrap mass spectrometer (Thermo Fisher Scientific; Waltham, MA) (iv) Negative ion mode analyses of free fatty acids and bile acids (C18-neg) were conducted using an LC-MS system comprised of a Shimadzu Nexera X2 U-HPLC (Shimadzu Corp.; Marlborough, MA) coupled to a Q Exactive hybrid quadrupole orbitrap mass spectrometer (Thermo Fisher Scientific; Waltham, MA). Samples were prepared for analysis using solid phase extraction.

**References**

1. Townsend MK, Clish CB, Kraft P, et al. Reproducibility of metabolomic profiles among men and women in 2 large cohort studies. *Clin Chem.* 2013;59(11):1657-1667.

2. Mascanfroni ID, Takenaka MC, Yeste A, et al. Metabolic control of type 1 regulatory T cell differentiation by AHR and HIF1-alpha. *Nat Med.* 2015;21(6):638-646.

3. O'Sullivan JF, Morningstar JE, Yang Q, et al. Dimethylguanidino valeric acid is a marker of liver fat and predicts diabetes. *J Clin Invest.* 2017.

4. Rowan S, Jiang S, Korem T, et al. Involvement of a gut-retina axis in protection against dietary glycemia-induced age-related macular degeneration. *Proc Natl Acad Sci U S A.* 2017;114(22):E4472-E4481.

5. Paynter NP, Balasubramanian R, Giulianini F, et al. Metabolic Predictors of Incident Coronary Heart Disease in Women. *Circulation.* 2018;137(8):841-853.
